# Supplementary material for: Validation of Four Thyroid Ultrasound Risk Stratification Systems in Patients with Hashimoto’s Thyroiditis; Impact of Changes in the Threshold for Nodule’s Shape Criterion
Source: Cancers (Basel). 2021 Sep 29;13(19):4900. doi: 10.3390/cancers13194900 (PMC8507673; doi:10.3390/cancers13194900)
Supplement: Supplementary file 1 [file cancers-13-04900-s001.zip › cancers-1365174-supplementary.pdf]

Supplementary Materials

Figure S1. ROC curve analysis of the evaluation of AP/T ratio on transverse plane in HT and non-HT groups; points of maximal ACC indicated on both curves

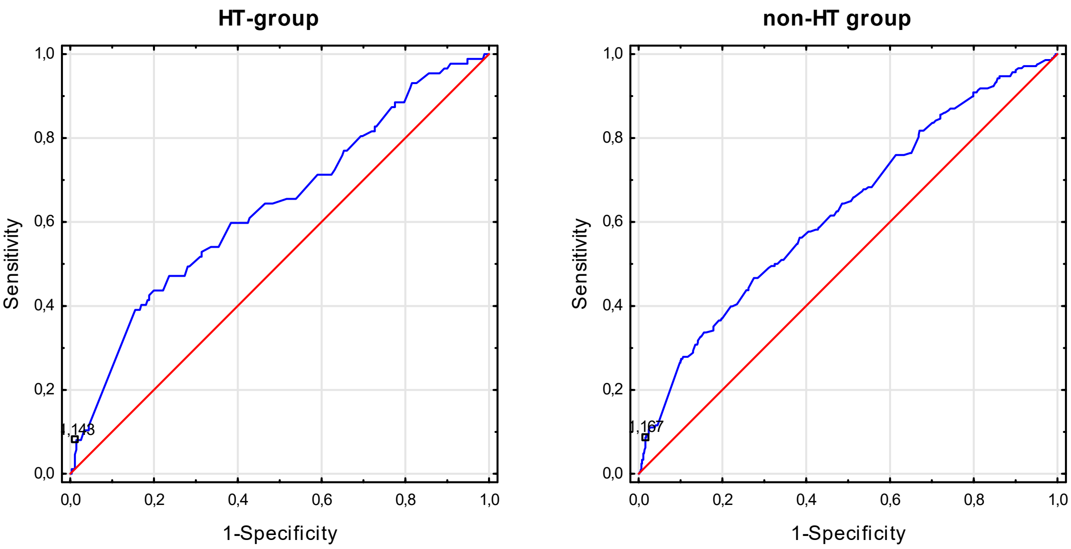

Figure S2. ROC curve analysis of the evaluation of AP/T ratio on longitudinal plane in HT and non-HT groups; points of maximal ACC indicated on both curves

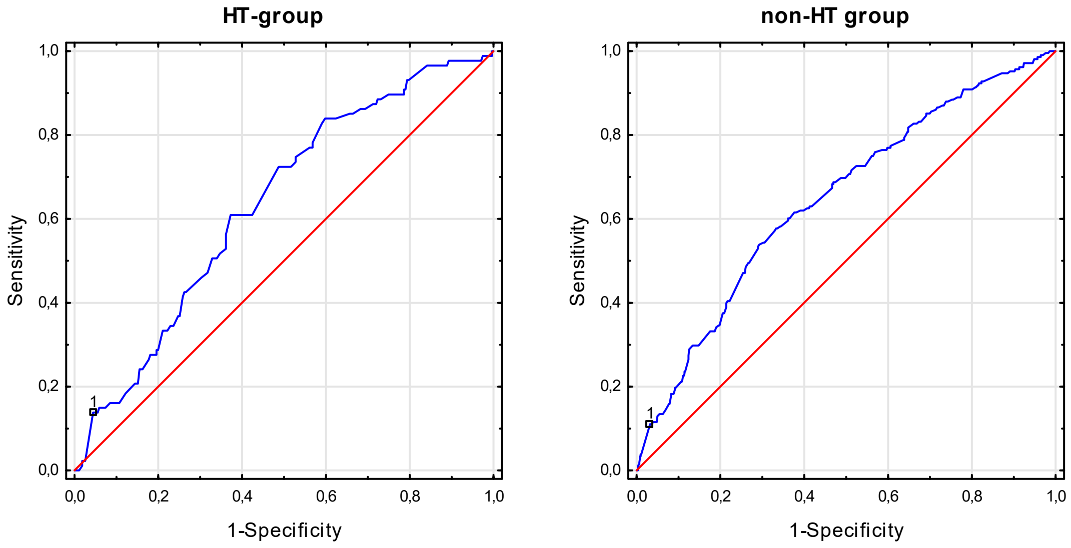

Table S1. Incidence of round nodules (with anteroposterior diameter equal to transverse diameter) in HT and non-HT group

The plane	No./% of round nodules					
	Benign			Malignant		
	HT group (271)	non-HT group (622)	p	HT group (87)	non-HT group (208)	p
transverse	30/11.1	33/5.3	0.0020	24/27.6	31/14.9	0.0108
longitudinal	5/1.8	13/2.1	0.8107	10/11.5	15/7.2	0.2284

Table S2. Data on the diagnostic efficacy of AP/T ratio evaluation in examined groups of nodules (HT and non-HT), with the threshold AP≥T or AP>T satisfied on any plane (transverse or longitudinal)

AP/T ratio	No/% of nodules	Ben./Mal. p	SEN	SPC	ACC	PPV (RoM)	NPV	LR+	OR CI95%/p
					**HT group**				
AP≥T	80/22.3	46/34 <0.0001	39.1	83.0	72.3	42.5	80.9	2.3	3.1 (1.8-5.4) <0.0001
AP>T	29/8.1	18/11 0.0743	12.6	93.4	73.7	37.9	76.9	1.9	2.0 (0.9-4.5). 0.0791
					**non-HT group**				
AP≥T	132/15.9	69/63 <0.0000	30.3	88.9	74.2	47.7	79.2	2.7	3.5 (2.4-5.1) <0.0001
AP>T	56/6.7	30/26 0.0001	12.5	95.2	74.5	46.4	76.5	2.6	2.8 (1.6-4.9) 0.0002

ACC – accuracy, AP – anteroposterior diameter, Ben. – benign lesion in histopathological outcome, CI - confidence intervals, HT – Hashimoto thyroiditis, LR+ - positive likelihood ratio, Mal. – thyroid malignancy in histopathological outcome, NPV – negative predictive value, OR – odds ratio, PPV – positive predictive value, RoM – risk of malignancy, SEN – sensitivity, SPC – specificity, T – transverse diameter

Table S3. Data on the diagnostic effectiveness of AP/T ratio evaluation (on transverse plane) in examined groups of nodules (HT and non-HT), in relation to nodule's size (largest diameter <1 cm and ≥1 cm)

AP/T ratio	Nodules <1 cm				Nodules ≥1 cm			
	**Ben. %**	**Mal. %**	**p**	**AUC (95%CI)**	**Ben. %**	**Mal. %**	**p**	**AUC (95%CI)**
				**HT group**				
AP≥T	28.6	43.3	0.2424	0.522 (0.402-0.701) 0.4975	14.0	36.8	0.0001	0.644 (0.561-0.727) 0.0007
AP>T	3.6	6.7	0.9511		4.5	14.0	0.0080	
AP/T≥1.14	3.6	6.7	0.9511		1.2	8.8	0.0065	
				**non-HT group**				
AP≥T	19.4	41.5	0.0374	0.651 (0.528-0.775) 0.0164	9.2	22.8	<0.0001	0.606 (0.557-0.656) <0.0001
AP>T	11.1	14.6	0.9052		4.1	10.8	0.0009	
AP/T≥1.17	5.6	12.2	0.5393		1.4	7.8	<0.0001	

AP – anteroposterior diameter, AUC - area under the receiver operating characteristic curve, Ben. – benign lesion in histopathological outcome, CI - confidence intervals, HT – Hashimoto thyroiditis, Mal. – thyroid malignancy in histopathological outcome, T – transverse diameter

Table S4. Results of multivariate logistic regression analysis in HT and non-HT groups

Sonographic feature	HT group			Sonographic feature	non-HT group		
	OR	CI95%	p		OR	CI95%	p
microcalcifications	13.2	4.4-40.1	<0.0001	irregular margins	9.0	5.1-15.8	<0.0001
irregular margins	6.1	2.7-13.7	<0.0001	microcalcifications	5.3	2.6-10.9	<0.0001
marked hypoechogenicity	4.2	1.8-9.8	0.001	marked hypoechogenicity	4.1	2.4-7.0	<0.0001
AP≥T	3.4	1.7-6.8	0.001	macrocalcifications	3.7	2.1-6.4	<0.0001
hypoechogenicity	2.8	1.3-6.0	0.008	solid echostructure	2.8	1.6-4.8	<0.0001
				hypoechogenicity	2.2	1.4-3.5	0.001
				AP≥T	2.1	1.1-4.0	0.030
				AP/T≥1.17	5.1	1.1-23.9	0.040

CI - confidence intervals, HT – Hashimoto thyroiditis

Table S5. Influence of modifications of the threshold for suspicious nodule's shape (from AP>T to AP≥T and AP/T≥1.14 in HT group or AP/T≥1.17 in non-HT group) on values of SEN, SPC, RoM and the percentage of nodules in the high risk category (changes expressed as percentage of values for AP>T threshold)

	**HT group**		**non-HT group**	
	**AP≥T**	**AP/T≥1.14**	**AP≥T**	**AP/T≥1.17**
	**EU-TIRADS threshold category: high risk (5)**			
% of nodules	32.6	-8.9	15.7	-7.3
SEN	11.2	-4.9	7.0	-0.8
SPC	-11.2	2.6	-4.7	2.8
RoM	-16.1	4.4	-7.4	7.2
	**K-TIRADS threshold category: high risk (5)**			
% of nodules	25.7	-5.9	21.5	-6.9
SEN	9.9	-6.1	11.3	-3.3
SPC	-4.7	0.4	-3.1	1.1
RoM	-12.2	0.0	-8.3	3.7
	**ACR-TIRADS threshold category: high risk (5)**			
% of nodules	39.7	-5.1	35.3	-10.1
SEN	18.6	-4.7	23.2	-5.7
SPC	-5.5	0.4	-3.2	0.9
RoM	-14.8	0.8	-9.2	4.7
	**ATA-guidelines threshold category: high risk (5)**			
% of nodules	26.7	-5.9	25.8	-6.9
SEN	12.0	-6.1	13.7	-2.4
SPC	-4.7	0.4	-3.8	1.3
RoM	-11.7	0.0	-9.7	4.8

AP – anteroposterior diameter, HT – Hashimoto thyroiditis, RoM – risk of malignancy, SEN – sensitivity, SPC – specificity, T – transverse diameter, TIRADS - thyroid imaging reporting and data systems
